# Electrochemiluminescence Sensor Based on CeO_2_ Nanocrystalline for Hg^2+^ Detection in Environmental Samples

**DOI:** 10.3390/molecules29010001

**Published:** 2023-12-19

**Authors:** Chunyuan Tian, Feiyan Tang, Wei Guo, Minggang Wei, Li Wang, Xuming Zhuang, Feng Luan

**Affiliations:** 1 College of Chemistry and Chemical Engineering, Yantai University, Yantai 264005, China; cytian@ytu.edu.cn (C.T.); tangfeiyan0807@163.com (F.T.); wmg18435221724@163.com (M.W.); fxhx@ytu.edu.cn (L.W.); xmzhuang@iccas.ac.cn (X.Z.); 2 Shandong Dyne Marine Biopharmaceutical Co., Ltd., Weihai 264300, China

**Keywords:** CeO_2_, electrochemiluminescence, mercury ions, aptamer, environment

## Abstract

The excessive concentration of heavy-metal mercury ions (Hg^2+^) in the environment seriously affects the ecological environment and even threatens human health. Therefore, it is necessary to develop rapid and low-cost determination methods to achieve trace detection of Hg^2+^. In this paper, an Electrochemiluminescence (ECL) sensing platform using a functionalized rare-earth material (cerium oxide, CeO_2_) as the luminescent unit and an aptamer as a capture unit was designed and constructed. Using the specific asymmetric matching between Hg^2+^ and thymine (T) base pairs in the deoxyribonucleic acid (DNA) single strand, the “T−Hg−T” structure was formed to change the ECL signal, leading to a direct and sensitive response to Hg^2+^. The results show a good linear relationship between the concentration and the response signal within the range of 10 pM–100 µM for Hg^2+^, with a detection limit as low as 0.35 pM. In addition, the ECL probe exhibits a stable ECL performance and excellent specificity for identifying target Hg^2+^. It was then successfully used for spiked recovery tests of actual samples in the environment. The analytical method solves the problem of poor Hg^2+^ recognition specificity, provides a new idea for the efficient and low-cost detection of heavy-metal pollutant Hg^2+^ in the environment, and broadens the prospects for the development and application of rare-earth materials.

## 1. Introduction

Electroluminescence (ECL) technology, as a new analytical method, has attracted much attention, combines the characteristics of both electrochemical and photochemical techniques and has the advantage of being easy to operate and portable [1,2]. This technology has low background signals and excellent sensitivity [3], due to the fact that the signal light source is not affected by the electrical energy of the excitation source, making it widely used in fields, such as environmental monitoring [4], biosensors [5], and immunoassays [6]. The emphasis on the development of ECL sensors lies in the construction of a sensing platform, in which the selection of ECL-active substances is the crucial element. Some research work has shown that traditional precious metal materials, such as gold [7,8], platinum [9], and ruthenium [10,11], have good electrical conductivity and ECL properties. However, their high prices limit their large-scale applications. Therefore, there is an urgent need to explore a simple, environmentally friendly, and low-cost ECL-active material to achieve the development of novel ECL signal amplification strategies.

Recent research developments have revealed that rare-earth nanomaterials have been widely used as attractive materials in ECL sensing analysis [12,13], due to their excellent luminescent properties and unique electron transfer properties of functional materials, which can enhance ECL signals by promoting electron transfer [14,15]. A rare-earth element terbium (Tb) metal complex was synthesized and constructed in an ECL sensor successfully with the ligand of pyridine-3-sulfonic acid (3-pSO_3_H) by Zhou’s team. When cadmium ions (Cd^2+^) were present in the environment, the ECL signal will be effectively quenched, achieving a sensitive response to Cd^2+^ in actual samples [16]. Yang et al. synthesized water-soluble nanoprobe iridium nanorods (Ir NRs) and further developed highly sensitive dual-signal Ir NRs@CdS quantum dots (QDs) with excellent luminescent properties, in which Ir NRs were used as the anodic emitter and CdS QDs as the cathodic emitter. Based on enzymatic reactions, a ratio-type change of ECL signals was generated, achieving a highly selective determination for ethyl paraoxon (EP) [17]. Babamiri et al. synthesized stable EuS nanocrystals and constructed ECL sensors. Combined with molecular imprinting technology, they explored a new method for a rapid and accurate response to human immunodeficiency virus HIV-1 [18]. It can be seen that rare-earth materials have the potential to build ECL sensing platforms. The development of ECL analysis strategies based on novel low-toxicity and environmentally friendly rare-earth-based inorganic semiconductor materials is receiving increasing attention. CeO_2_ NPs, being important rare-earth-based inorganic semiconductor materials, have obvious chemical properties, optical, magnetic properties, and good photochemical stability. At present, there is still little research on these kinds of materials in the field of ECL [19,20].

As one of the transitional heavy-metal elements, mercury pollution incidents occur frequently around the world [21]. Due to the nondegradability of mercury ions (Hg^2+^), inappropriate emissions can lead to their accumulation and long-term existence in the environment, and pollution of aquatic ecosystems can even affect human health through the food chain [22,23]. It has been reported that the ingestion of trace amounts of Hg^2+^ can cause varying degrees of damage to the central nervous system, kidneys, and brain [24,25]. Therefore, the rapid and sensitive determination of Hg^2+^ in environmental samples is of great significance. In previous research, traditional analytical methods, such as atomic absorption spectrometry (AAS) [26], mass spectrometry (MS) [27], and inductively coupled plasma (ICP) [28], have been widely applied for the determination of Hg^2+^; however, they usually require professional personnel to operate and some techniques do not meet the international standard for the detection of mercury in real samples. In recent years, researchers have focused on improving the sensitivity of detection techniques and developing new inspection methods to identify and detect trace amounts of Hg^2+^ [29,30,31].

It is worth noting that ligand recognition technology has developed rapidly in the field of analysis and detection due to its stable, inexpensive, and easy modification of deoxyribonucleic acid (DNA) in many analysis strategies [32,33]. In 2004, it was first discovered that thymine (T) in the nucleic acid sequence can preferentially and specifically bind to Hg^2+^ over cytosine (A), resulting in a mismatch of base pairs to form a “T−Hg−T” structure [34].

**Figure 1:**
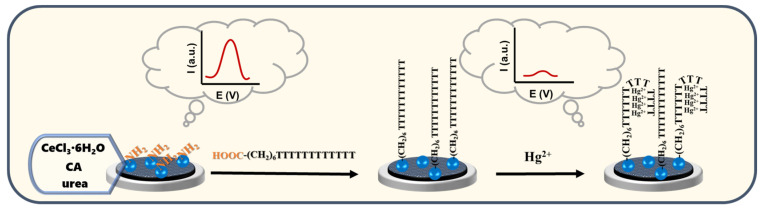
Construction of the ECL sensor and mechanism diagram for detecting Hg^2+^.

Based on this recognition mechanism, this work utilized a hydrothermal method to synthesize amino-modified cerium oxide nanomaterials (CeO_2_ NPs) and constructed an Hg^2+^ recognition platform by combining amide bonds with T base-containing aptamers. When Hg^2+^ was present in the solution, T-rich aptamers formed a stem–ring structure due to “T−Hg−T” asymmetric pairing, specifically capturing and quantitatively responding to Hg^2+^ within the concentration range of 10 pM–100 µM (Figure 1). Owing to the highly specific binding ability between Hg^2+^ and aptamers, other ions coexisting in a complex sample do not interfere with its detection. Fish and shrimp samples were then tested using the proposed ECL sensor with excellent correlations, suggesting that the proposed sensor is of great promise in Hg^2+^ detection at low concentrations in the environment. Compared with other ECL methods to detect Hg^2+^, our method is more direct and simpler. 

## 2. Results

### 2.1. Morphological Characterization

**Figure 2:**
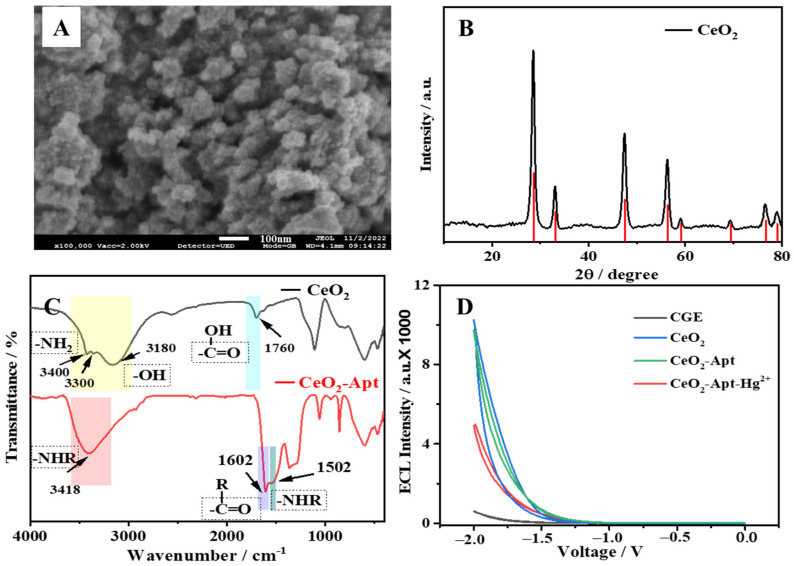
(**A**) SEM image of CeO_2_. (**B**) XRD spectrum of CeO_2_ (black line) and XRD standard characteristic diffraction peak of CeO_2_ (red line). (**C**) FT-IR spectra of CeO_2_ (black line) and CeO_2_-Apt (red line). (**D**) ECL spectra of the bare GCE (black line), CeO_2_ (blue line), CeO_2_-Apt (green line), and CeO_2_-Apt-Hg^2+^ (red line).

The morphology of CeO_2_ NPs was characterized by using a scanning electron microscope (SEM, JEOL, Tokyo, Japan), and it can be clearly observed that the material was formed by the agglomeration of small particles with a particle diameter of 100 nm, as shown in Figure 2A. The analysis results of elements in the dispersive spectrometer (EDS) mapping diagrams of Figure S1 prove that Ce and O were the main constituent elements, and N was uniformly distributed on the surface of CeO_2_. In addition, the X-ray diffractometer (XRD) spectrum of CeO_2_ (Figure 2B) could further confirm the successful synthesis of the CeO_2_ material by comparison with the XRD standard card.

The changes in the surface groups of the CeO_2_ and CeO_2_-Apt materials were analyzed by using Fourier transform infrared (FT-IR) spectroscopy. As shown in Figure 2C, the existence of a stretching vibration peak of N–H (υ(N–H)) can be clearly found at 3200–3500 cm^−1^ in CeO_2_ (black line). Compared with reference [35], it can be proven that amino groups (-NH_2_) were rich in the CeO_2_ material surface. Meanwhile, the FT-IR peaks situated at 3180 cm^−1^ for υ(O–H) and 1760 cm^−1^ for υ(C=O) jointly prove that the surface of CeO_2_ also contained a handful of carboxylic groups (-COOH), which can be considered to be induced from the raw material CA in the synthesis operation. A secondary amine bond of υ(N–H) was observed at 1502 cm^−1^ and an amide bond υ(CON–R) at 3418 cm^−1^ of CeO_2_-Apt (red line) in conformity with previous reports [36], which demonstrated the successful binding of the aptamer. Further, when comparing the ECL performance generated by each fabrication step of the sensor platform, as shown in Figure 2D, it can be clearly seen that the synthesized CeO_2_ material had excellent ECL strength and the binding of the aptamers generated a certain steric hindrance resulting in a slight decrease in the ECL emission intensity. When target molecules of Hg^2+^ were present in the environmental sample, the specific capture led to a further increase in steric hindrance, which significantly quenched the ECL signal, thus achieving recognition and detection of Hg^2+^.

### 2.2. Optimization of Experimental Conditions

To obtain the best ECL performance of this Hg^2+^ ECL sensor, the pH of the sensing environment was first optimized. Coreactant solutions with different pH values (3.4, 4.4, 5.4, 6.4, 7.4, 8.4, 9.4, and 10.4) were selected for ECL testing, as shown in Figure S2A. The results show that CeO_2_ had the best ECL emission when the test environment pH increased to 7.4, which conformed to the pH in most natural environments. Thus, the following ECL experiments were performed at this pH. Subsequently, the CeO_2_ drop-coating concentration during the construction of the sensing platform was optimized, in which the concentration increased from 0.1 to 3 mg·mL^−1^, as shown in Figure S2B. The experimental results indicate that too small or too large of a concentration did not facilitate the ECL emission of CeO_2_. Therefore, a concentration of 1 mg·mL^−1^ was selected for drop coating in subsequent tests.

### 2.3. ECL Mechanism of CeO_2_

There was a further analysis of the possible ECL mechanism of the CeO_2_ material. Charge injection reduced CeO_2_ on the GCE surface to negatively charged radicals (CeO_2_^•−^) under an initial negative potential. At the same time, the coreactant S_2_O_8_^2−^ in the solution also obtained electrons, generating the free radicals (SO_4_^•−^) and SO_4_^2−^. The two free-radical ions collided and exchanged energy, and the high-energy excited states of CeO_2_ (CeO_2_*) and SO_4_^2−^ were produced. However, CeO_2_* was unstable and returned to the ground state to release light energy. The relationship formula was as follows:

CeO_2_ + e^−^ → CeO_2_^•−^ (1)

S_2_O_8_^2−^ + e^−^ → SO_4_^2−^ + SO_4_^•−^ (2)

CeO_2_^•−^ + SO_4_^•−^ → CeO_2_* + SO_4_^2−^ (3)

CeO_2_* → CeO_2_ + *hυ* (4)

Adapters utilized -COOH groups on the surface to bind CeO_2_ through amide bonds. When Hg^2+^ exists in the environmental sample, the T base pairs in the aptamer can specifically capture Hg^2+^ to form a “T−Hg−T” structure, leading to the bending of the aptamer structure. Doing that, the ECL signal was quenched due to the fact that the electron transfer was blocked as the structure changed.

### 2.4. Response of the ECL Sensor to Hg^2+^

**Figure 3:**
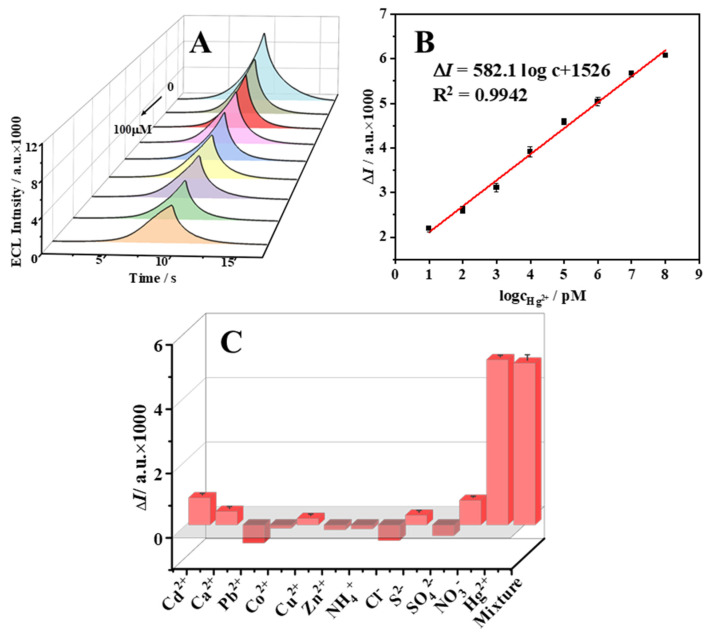
(**A**) ECL signals measured at different concentrations of Hg^2+^ (0–100 µM). (**B**) Logarithmic calibration curve between ECL signal change value and Hg^2+^ concentration. (**C**) Comparison of ECL signal changes generated by different interfering ions and the mixed solution at the same concentration.

For the purpose of a quantitative assessment of the Hg^2+^ concentration in the environmental sample, this sensor was designed for the specific identification of different concentrations (100 µM, 10 µM, 1 µM, 100 nM, 10 nM, 1 nM, 100 pM, and 10 pM) of Hg^2+^ under optimal experimental conditions. When experimentally analyzed, the ECL response signals decreased with increasing Hg^2+^ concentrations in the bare solution, as shown in Figure 3A, which was consistent with the above reaction mechanism. The linear relationship and equation established between the concentration logarithmic value of the recognition unit of Hg^2+^ and the quenching value ΔI of ECL are shown in Figure 3B. The fitted linear equation was ΔI = 582.1 log c + 1526, R^2^ = 0.9942. A good linear relationship with a limit of detection (LOD, S/N = 3) as low as 0.35 pM was indicated. Compared to the techniques for detecting Hg^2+^ reported in other references, as shown in Table S1, the ECL sensor was not only simple to operate but also had a wide linear range and excellent LOD based on CeO_2_, which has certain superiority in identifying trace amounts of Hg^2+^.

To verify specific selectivity, several common interference ions in the environment were utilized for selective testing of the sensor. The change signals of different ions are shown in Figure 3C. Significant ECL signal quenching was exhibited for Hg^2+^ and solutions containing Hg^2+^, and the response for other ions was negligible, which indicated that the sensor only had a specific response to solutions containing Hg^2+^. Thus, the results of the experiment demonstrate the reliability and accuracy of this experimental strategy and greatly broaden its application prospects.

### 2.5. Stability of the ECL Sensor

**Figure 4:**
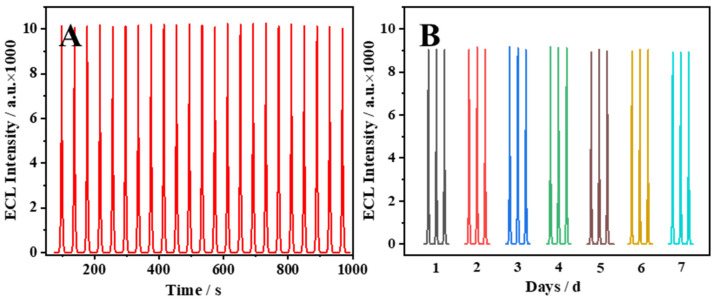
(**A**) ECL scan signal obtained by 23 consecutive cycles of CeO_2_/GCE in the sensors. (**B**) ECL signal of one CeO_2_-modified electrode in one week under the same experimental conditions.

The stability of the ECL sensing platform constructed with modified electrodes by CeO_2_ NPs was tested. Consecutive cycles of 23 cycles were tested in PBS solution containing 0.1 M K_2_S_2_O_8_ of one electrode to verify the ECL stability. As shown in Figure 4A, one electrode exhibited a strong and stable ECL signal in the same ECL test. When verifying short-term stability, the 7-day stability of the same modified electrodes was measured, as shown in Figure 4B. The ECL performance of the CeO_2_-modified electrode hardly changed within 7 days, which demonstrates the excellent ECL stability of CeO_2_/GCE.

### 2.6. Detection of Hg^2+^ in Actual Samples

Spiking tests were used to verify the application potential of this ECL sensor in actual environmental samples (fish and shrimp). The results are shown in Table 1. The spiked recovery rate was between 82.99% and 105.0%, and the relative standard deviation (RSD) was less than 2.5%. Evidently, it can be utilized for the direct and accurate determination of Hg^2+^ in subsequent actual environmental samples based on a satisfactory spiked recovery effect. 

## 3. Materials and Methods

### 3.1. Reagents and Chemicals

Cerium nitrate hexahydrate (CeCl_3_·6H_2_O, AR), urea (AR), citric acid (CA, AR), hydrogen peroxide (H_2_O_2_, AR), potassium persulfate (K_2_S_2_O_8_, AR), sodium phosphate dibasic dodecahydrate (Na_2_HPO_4_·2H_2_O, AR), potassium dihydrogen phosphate (KH_2_PO_4_, AR), N-hydroxysuccinimide (NHS, AR), 1-(3-Dimethylaminopropyl)-3-ethylcarbodiimide (EDC, AR), and mercuric chloride (HgCl_2_, AR) were obtained from Aladdin Biochemical Technology Co., Ltd. (Shanghai, China). Cadmium nitrate (Cd(NO_3_)_2_, AR), sodium chloride (NaCl, AR), sodium nitrate (NaNO_3_, AR), barium chloride (BaCl_2_, AR), lead nitrate (Pb(NO_3_)_2_, AR), cobalt chloride (CoCl_2_, AR), sodium sulfate decahydrate (Na_2_S·9H_2_O, AR), and sodium sulfate (Na_2_SO_4_, AR) were purchased from Sinopharm Chemical Reagents Co., Ltd. (Tianjin, China). The Hg^2+^ aptamer (5′-COOH-(CH_2_)_6_-TTTTTTTTTTTT-3′) was synthesized by Shanghai Sangon Biotechnology Co., Ltd. (Shanghai, China). The reagents used in this study were not subjected to purification treatment unless otherwise specified. The solutions involved were all prepared with ultrapure water (18.25 MΩ cm).

### 3.2. Apparatus

The ECL signals were measured by using a type of MPI-E ECL Analysis System (Xi’An Remax Electronic Science & Technology Co., Ltd., Xi’an, China). SEM images and EDS element mapping images were obtained by using JSM-7900F (JEOL, Tokyo, Japan). XRD spectrum was acquired by using XRD-Smart Lab (3 kW, Smart Lab, Tokyo, Japan). FT-IR spectrum was obtained by using IR Affinity-1S (Shimadzu, Shanghai, China).

### 3.3. Synthesis of CeO_2_ NPs

CeO_2_ was synthesized according to previous reports [31]. Briefly, 0.02 g of CA, 0.1 g of CeCl_3_·6H_2_O, and 0.16 g of urea were fully mixed and completely dissolved in 25 mL of ultrapure water, and subsequently, 0.15 mL of H_2_O_2_ (30%) was added dropwise. The above-mixed solution was heated to 180 °C for 20 h in 45 mL of polytetrafluoroethylene liner. The suspension containing white solids was washed with water and alcohol three times after the reaction was completed and cooled to room temperature. The obtained white solids of CeO_2_ NPs were dried overnight at 60 °C and stored at 4 °C for subsequent experiments.

### 3.4. Synthesis of CeO_2_-Apt

First, 1 mL Hg^2+^ aptamer (0.1 mM) was added to the mixed solution including 2 mL EDC (25 mg·mL^−1^) and 2 mL NHS (12 mg·mL^−1^) and stirring continued for 1.5 h at room temperature to activate the carboxyl group (-COOH) on the surface of the aptamer. Soon afterward, 1 mg of CeO_2_ was added and fully reacted for 2 h at room temperature to the above solution. Hence, CeO_2_ modified with the aptamer (CeO_2_-Apt) was obtained by centrifuging with ultrapure water three times and dried at 60 °C, Finally, the sample was stored at 4 °C for further use.

### 3.5. Construction of the Hg^2+^ ECL Sensor

A total of 1 mg CeO_2_-Apt powder was dissolved in 1 mL ultrapure water to prepare the material solution (1 mg·mL^−1^). Then, 5 µL was dropwise added to the clean surface of the glassy carbon electrode (GCE). The working electrode CeO_2_-Apt/GCE was obtained after drying at room temperature; the counter electrode was a platinum (Pt) wire electrode; and the reference electrode was an Ag/AgCl electrode (the concentration of KCl in the filled liquid was 3 M). The working voltage range of the electrochemical analyzer was set to be −2.0–0 V; the scanning rate was 100 mV s^−1^; the photomultiplier tube (PMT) was 800 V; and the coreactant to be selected was 0.1 M K_2_S_2_O_8_ (5 mL) solution that was confected by 0.1 M phosphate buffer solution (PBS, pH = 7.4). We performed an ECL response test on a series of 100 µL concentrations of Hg^2+^ at 10 pM, 100 pM, 1 nM, 10 nM, 100 nM, 1 µM, 10 µM, and 100 µM. Based on ECL signals to construct a linear standard curve between different Hg^2+^ concentrations and ECL quenching values, Δ*I* (Δ*I* = *I*_0_ − *I*, *I*_0_ is the blank signal of the ECL without the addition of Hg^2+^ to be tested, and I is the ECL signal value after the addition of different concentrations of Hg^2+^) was obtained from the test.

### 3.6. Selective Testing

Several common anions and cation ions (Cd^2+^, Ca^2+^, Pb^2+^, Co^2+^, Cu^2+^, Zn^2+^, NH_4_^+^ Cl^−^, S^2−^, SO_4_^2−^, and NO_3_^−^) in the environment were selected as interfering substances and prepared at 1 µM. After that, we added the above-interfering ions and a mixed solution with them and the same concentration of Hg^2+^ and collected the response values of the different solutions.

### 3.7. Actual Sample Test

Fish and shrimp were selected as actual samples to evaluate the practical application potential of this Hg^2+^ ECL sensor. The fish and shrimp used in this study were purchased from Yantai University Market. The standard addition method was used to detect the content of Hg^2+^ in fish and shrimp. First, fish and shrimp meat were subjected to pretreatment, and 1.0 g of meat was washed and shredded before being added in 10 mL of ultrapure water. Then, the above solutions were broken by using a cell disruptor for 30 min, followed by centrifugation to extract the supernatant. Then, a filter membrane with a pore diameter of 0.22 µm was used for further filtration. The solutions were divided into equal parts, and spiked sample solutions were prepared with concentrations of 100 pM, 100 nM, and 100 µM of Hg^2+^, and the spiked recovery experiments were carried out under the same conditions.

## 4. Conclusions

In the present study, a simple-to-operate and environmentally friendly ECL sensor was constructed based on the inexpensive and easily synthesized CeO_2_ NPs. The results indicate that CeO_2_ NPs were the ideal ECL emitter with a strong and stable ECL performance. The established Hg^2+^ sensing platform achieved an accurate determination of Hg^2+^ concentrations within the range of 10 pM–100 μM, with an LOD as low as 0.35 pM. Finally, the ECL sensor was successfully applied for the detection of trace Hg^2+^ in actual samples in the environment. This strategy greatly reduced the research costs, improved the detection sensitivity and response speed, and also provided the possibility for the development of rare-earth materials in the ECL field. 

## Figures and Tables

**Table 1:** Determination of Hg^2+^ in actual environmental samples by the ECL sensor (*n* = 3).

Samples	Added (pM)	Found (pM)	Recovery (%)	RSD (%)
Fish	1 × 10^2^	0.8299 × 10^2^	82.99	0.81
	1 × 10^5^	1.050 × 10^5^	105.0	1.4
	1 × 10^8^	0.9258 × 10^8^	92.58	1.7
Shrimp	1 × 10^2^	0.8831 × 10^2^	88.31	0.7
	1 × 10^5^	0.8730 × 10^5^	87.30	2.5
	1 × 10^8^	0.8551 × 10^8^	85.51	0.73

## Data Availability

The data are available upon reasonable request.

## Supplementary Materials

The following supporting information can be downloaded at: https://www.mdpi.com/article/10.3390/molecules29010001/s1, Figure S1: EDS mapping images of (A) CeO_2_ (B) Ce (C) O (D) N; Figure S2: (A) Optimization of pH of ECL sensing environment (B) The concentration of the CeO_2_ solution dripped onto the electrode surface; Table S1: Comparison between CeO_2_ ECL sensor and other methods for detecting Hg^2+^. References [37,38,39,40] are cited in the Supplementary Materials.
