# Supplementary material for: Electrochemiluminescence Sensor Based on CeO2 Nanocrystalline for Hg2+ Detection in Environmental Samples
Source: Molecules. 2023 Dec 19;29(1):1. doi: 10.3390/molecules29010001 (PMC10779929; doi:10.3390/molecules29010001)
Supplement: Supplementary file 1 [file molecules-29-00001-s001.zip › molecules-2755282-supplementary.pdf]

Supporting Information

Electrochemiluminescence Sensor Based on CeO_2 Nanocrystalline for Hg^{2+} Detection in Environment Samples

Chunyuan Tian [1], Feiyan Tang [1], Wei Guo [2,*], Minggang Wei [1], Li Wang [1], Xuming Zhuang [1] and Feng Luan [1,*]

[1] College of Chemistry and Chemical Engineering, Yantai University, Yantai 264005, China

[2] Shandong Dyne Marine Biopharmaceutical Co., Ltd, Weihai 264300, China

* Correspondence: guodawei0298@163.com (W.G.); fluan@sina.com (F.L.)

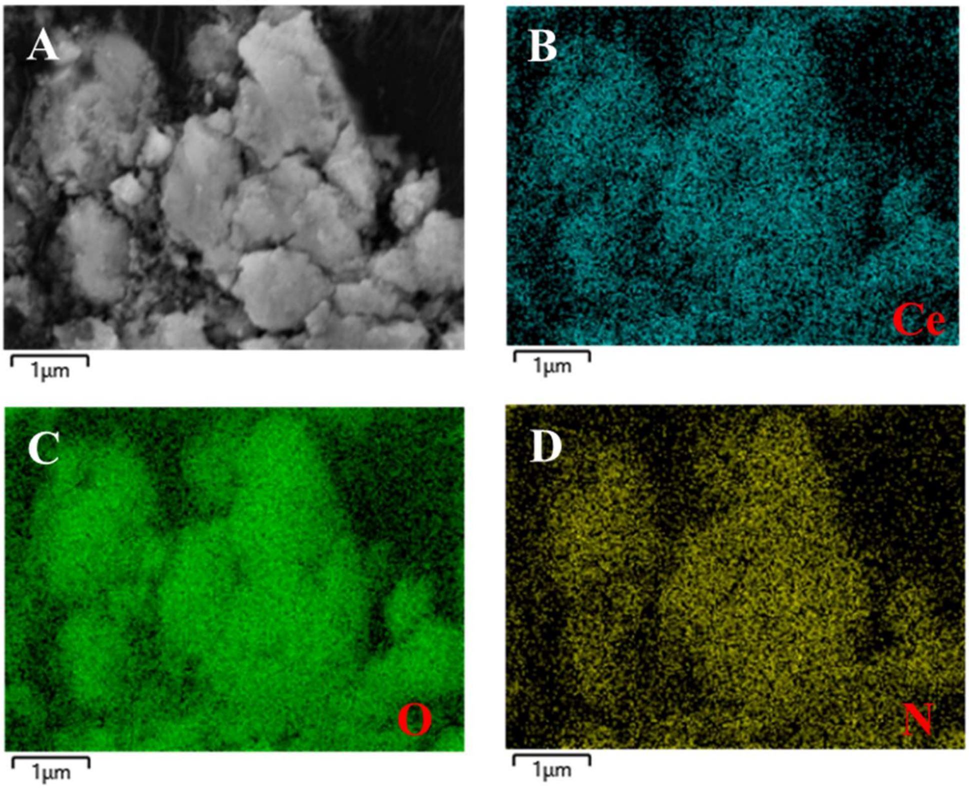

Figure S1. EDS-Mapping images of (A) CeO_2; (B) Ce; (C) O; (D) N.

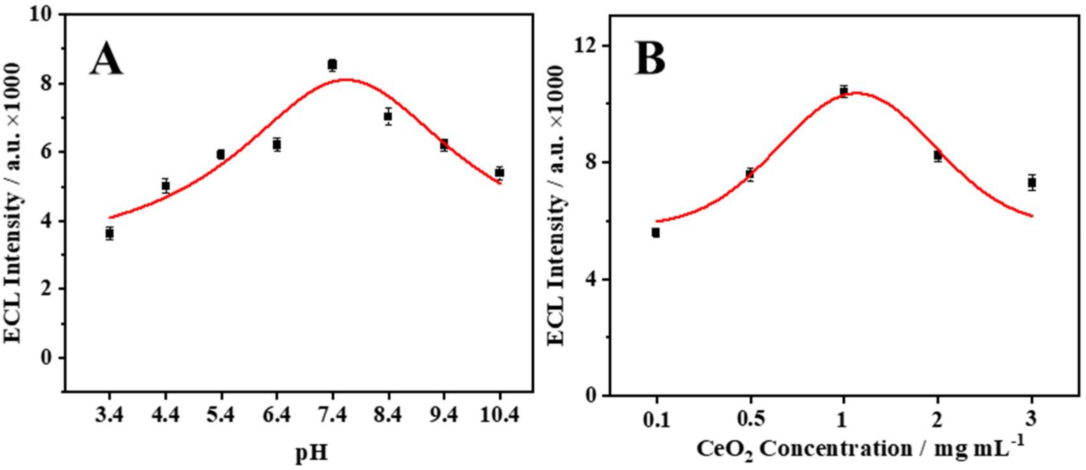

Figure S2. (A) Optimization of pH of ECL sensing environment; (B) The concentration of the CeO_2 solution dripped onto the electrode surface.

Table S1. Comparison between CeO_2 ECL sensor and other methods for detecting Hg^{2+}.

Probe materials	Testing method	Linear range	LODs	Ref.
MoS_2@Au NPs	FL	0.02-1 μM	9.41 nM	1
HMA[a]/Eu^{3+}-CdS	PEC[b]	0.1-1 μM	0.067 μM	2
CuS HNSs[c]	UV-Vis[d]	50 pM-400 nM	50 pM	3
Au NPs/PLL[e]-BP[f]	EC[g]	1-500 nM	0.14 nM	4
CeO_2 NPs	ECL	10 pM-100μM	0.35 pM	This work

[a] hydrophobically modified alginate (HMA)
[b] photoelectrochemistry (PEC)
[c] hollow nanospheres (HNSs)
[d] ultraviolet–visible (UV-Vis)
[e] poly-L-lysine (PLL)
[f] black phosphorus (BP)
[g] electrochemistry (EC)

Reference

1. Liu, C.; Tuffour, A.; Liao, J.H.; Li, M.L.; Lv, Q.X.; Zhou, D.R.; Gao, L. Highly sensitive detection of Hg^{2+} using molybdenum disulfide-DNA sensors. *Chem. Phys.* **2020,** 534, 110758.
2. Bu, Y.W.; Wang, K.; Yang, X.Y.; Nie, G.M. Photoelectrochemical sensor for detection Hg^{2+} based on in situ generated MOFs-like structures. *Anal. Chim. Acta,* **2022,** 1233, 340496.
3. Fang, Y.M.; Zhang, Y.; Cao, L.G.; Yang, J.Z.; Hu, M.H.; Pang, Z.L.; He, J.H. Portable Hg^{2+} Nanosensor with ppt Level Sensitivity Using Nanozyme as the Recognition Unit, Enrichment Carrier, and Signal Amplifier. *ACS Appl. Mater. Interfaces,* **2020,** 12(10), 11761- 11768.
4. Zhou, J.; Wei, D.; Hu, C.C.; Li, S.; Ma, L.; Chen, Z.; Deng, Y. A simple label-free electrochemical aptamer sensor for detection of Hg^{2+} based on black phosphorus. *Mater. Express,* **2022,** 12(6), 802- 809.
